# Amplification of Endoplasmic Reticulum Stress via Inhibiting Lipid Droplet Formation to Enhance Chemodynamic Immunotherapy

**DOI:** 10.1002/advs.75626

**Published:** 2026-05-10

**Authors:** Huilan Cai, Shaoru Zhuang, Yang Zhu, Rujiang Ao, Meili Yu, Tingting Cui, Jun Wang, Xuegang Niu, Hongwei Huang, Shanshan Peng, Yu He, Lisen Lin, Huanghao Yang

**Affiliations:** ^1^ New Cornerstone Science Laboratory MOE Key Laboratory for Analytical Science of Food Safety and Biology College of Chemistry Fuzhou University Fuzhou China; ^2^ Department of Neurosurgery Neurosurgery Research Institute The First Affiliated Hospital of Fujian Medical University Fuzhou China

**Keywords:** chemodynamic immunotherapy, endoplasmic reticulum stress, hydroxyl radicals, immunogenic cell death, lipid droplets

## Abstract

Chemodynamic generation of hydroxyl radicals (•OH) from endogenous hydrogen peroxide (H_2_O_2_) in the endoplasmic reticulum (ER) is promising to initiate cancer immunotherapy via ER stress‐mediated immunogenic cell death (ICD). However, the sprout of lipid droplets (LDs) is elevated under ER stress, which may limit chemodynamic immunotherapy due to the ability of LDs to reduce the production of ER stress‐inducing 4‑hydroxynonenal (4‐HNE, a lipid peroxidation (LPO) byproduct) by sequestering polyunsaturated fatty acids (PUFAs) and to block “eat‐me” signals by recruiting calreticulin (CRT). Here, an ER‐targeted chemodynamic nanoagent (denoted as TCBQ/iLD‐ER NPs) is reported for LDs downregulation‐enhanced chemodynamic immunotherapy. After uptake by tumor cells, TCBQ/iLD‐ER NPs comprising •OH‐generating tetrachloro‐1,4‐benzoquinone (TCBQ) and A‐922500 (an LDs formation inhibitor, defined as iLD) preferentially accumulate in the ER, where TCBQ reacts with high levels of ER H_2_O_2_ to yield •OH accompanied by iLD release. •OH‐triggered LPO of PUFAs not only causes cancer cell death, but also produces 4‐HNE to provoke ER stress‐mediated ICD. Intriguingly, iLD inhibits LDs formation and thereby reduces the sequestration of PUFAs as well as the recruitment of CRT by LDs, enabling improved chemodynamic immunotherapeutic efficacy. This study highlights a versatile strategy to enhance chemodynamic immunotherapy by modulating intracellular LDs.

## Graphical abstract

An endoplasmic reticulum (ER)‐targeted chemodynamic nanoagent (TCBQ/iLD‐ER NPs) comprising •OH‐generating tetrachloro‐1,4‐benzoquinone (TCBQ) and lipid droplets (LDs) formation inhibitor (iLD) is developed for chemodynamic immunotherapy of tumors. After ingestion by cancer cells, TCBQ/iLD‐ER NPs preferentially accumulate in the ER and react with ER H_2_O_2_ to yield •OH accompanied by iLD release, enabling LDs downregulation‐enhanced chemodynamic immunotherapeutic efficacy.

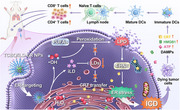

## 1 Introduction

Cancer immunotherapy aiming to activate the organism's immune system against tumor metastasis and recurrence has achieved significant progress in recent years [1, 2, 3, 4]. However, low immunogenicity of cancerous cells makes immunotherapeutic efficacy somewhat unsatisfactory [5, 6, 7]. Therefore, it is necessary to seek approaches to effectively stimulate anti‐tumor immune responses. Immunogenic cell death (ICD), is a specific type of cell death, enabling tumor cells to convert from non‐immunogenic to immunogenic [8, 9]. ICD drives the release of damage‐associated molecular patterns (DAMPs), including calreticulin (CRT), high mobility group box 1 (HMGB1), and adenosine triphosphate (ATP), which could promote the maturation of dendritic cells (DCs) and the ensuing anti‐tumor immunity [10, 11, 12]. Notably, reactive oxygen species (ROS) has been reported to trigger not only oxidative damage in cancer cells but also endoplasmic reticulum (ER) stress‐mediated ICD [13, 14, 15, 16]. Particularly, chemodynamic therapy (CDT), which utilizes chemical reactions to form highly cytotoxic ROS from endogenous hydrogen peroxide (H_2_O_2_), has emerged as a promising approach to initiate cancer immunotherapy [17, 18, 19]. As known, ROS possesses the ability to oxidize lipids, especially polyunsaturated fatty acids (PUFAs), and subsequently induce lipid peroxidation (LPO) [20, 21, 22]. On the one hand, ROS‐triggered LPO can cause cancer cell death through the disruption of cell membrane [23, 24]; on the other hand, the LPO byproduct 4‑hydroxynonenal (4‐HNE) could induce ER stress because of its capability to interfere with protein folding through direct modification of ER proteins, provoking ER stress‐mediated ICD [25, 26]. Importantly, the ER H_2_O_2_ maintains at a relatively high level, as it is continuously generated during protein folding in the ER lumen [27, 28, 29, 30]. Thus, it is conceivable that chemodynamic agents targeted to the ER can generate more adequate ROS in situ to better induce ER stress‐mediated chemodynamic immunotherapy.

Currently, ROS‐generating CDT agents are increasingly being employed in tumor immunotherapy, and the ER has been regarded as an important immunotherapeutic target [31, 32]. ER is a vital organelle involved in diverse biological processes, and ROS can disrupt ER homeostasis and result in ER stress [33, 34, 35]. However, ER stress could stimulate diacylglycerol‐acyltransferase 1 (DGAT1)‐mediated sprout of lipid droplets (LDs) from the ER membrane in large amounts [36, 37, 38]. Notably, DGAT1 is an ER‐resident enzyme involved in triacylglycerol (TAG) biosynthesis, and TAG is a common component within the LDs core surrounded by a phospholipid monolayer [39, 40, 41]. Importantly, DGAT1 drives the transformation of PUFAs to TAG that is then packaged into LDs, thereby protecting PUFAs from harmful peroxidation and reducing ER stress elicited by the LPO byproduct 4‐HNE [42, 43, 44]. Meanwhile, LDs can also recruit CRT and thus block the transfer of CRT to the cell membrane, limiting the exposure of “eat‐me” signals on the plasma membrane [45, 46]. Conceivably, the presence of LDs may limit both the chemodynamic cytotoxicity and immunity‐activating effect of CDT agents. Therefore, inhibiting the formation of LDs appears to be a promising method for the enhancement of chemodynamic immunotherapy. Nevertheless, to our knowledge, there is no report on the modulation of LDs formation to intensify chemodynamic immunotherapeutic efficacy.

**SCHEME 1:**
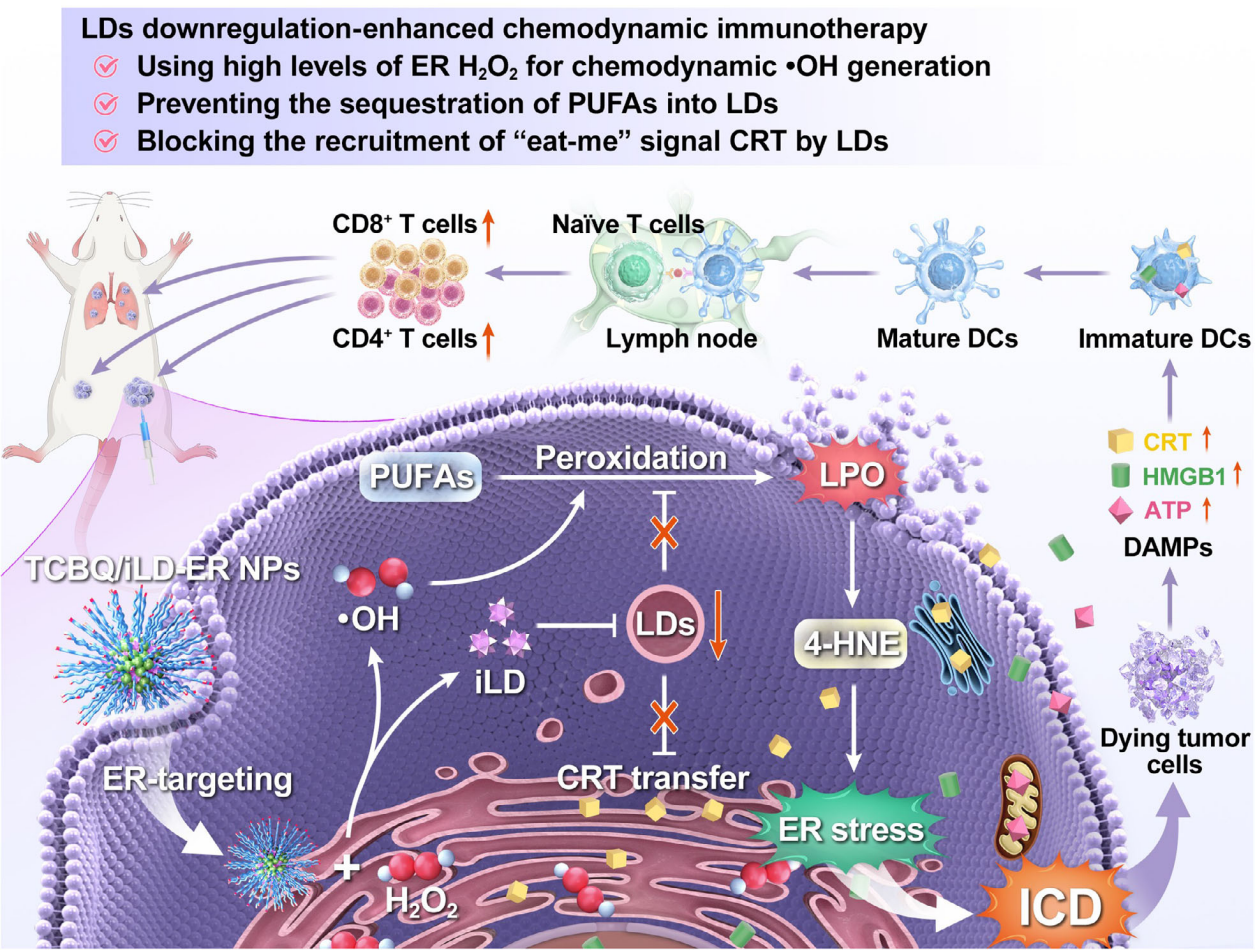
Schematic representation of TCBQ/iLD‐ER NPs for enhanced chemodynamic immunotherapy via inhibiting LDs formation.

Herein, we developed an ER‐targeted CDT nanoagent to achieve enhanced chemodynamic immunotherapy of tumors by suppressing LDs formation (Scheme 1). The CDT nanoagent (denoted as TCBQ/iLD‐ER NPs) was fabricated by co‐encapsulating ROS‐generating tetrachloro‐1,4‐benzoquinone (TCBQ) and A‐922500 (a DGAT1 inhibitor able to block LDs formation, defined as iLD) via a nanoprecipitation method with ER‐targeting moiety‐modified amphiphilic polymer as encapsulation matrix. Notably, hydrophobic TCBQ could be converted into water‐soluble trichlorohydroxy‐1,4‐benzoquinone (TrCBQ‐O^−^) after reacting with H_2_O_2_ at physiological pH 7.4, accompanied by the production of hydroxyl radicals (•OH) and the degradation of TCBQ/iLD‐ER NPs to release iLD capable of inhibiting the enzymatic activity of DGAT1 as well as DGAT1‐mediated LDs formation. After ingestion by cancerous cells, TCBQ/iLD‐ER NPs preferentially accumulated in the ER, followed by reaction with high levels of ER H_2_O_2_ to yield plentiful •OH that could oxidize PUFAs and subsequently induce LPO. On the one hand, ROS‐triggered LPO caused cancer cell death, triggering chemodynamic toxicity against tumor cells; on the other hand, the LPO byproduct 4‐HNE induced ER stress, provoking ER stress‐mediated ICD. Interestingly, the released iLD blocked LDs formation and reduced the sequestration of PUFAs as well as the recruitment of CRT by LDs, enabling TCBQ/iLD‐ER NPs to efficiently exert their chemodynamic immunotherapeutic effects. Ultimately, CDT‐induced ICD of cancer cells accelerated the release of DAMPs to promote DCs maturation, followed by enhanced T cells activation, resulting in a systemic anti‐tumor immune response. The TCBQ/iLD‐ER NPs successfully suppressed primary and metastatic tumors, dampened the lung metastasis of cancerous cells, and prolonged the life span of tumor‐bearing mice. This work highlights a promising approach to boost chemodynamic immunotherapy by modulating the levels of intracellular LDs.

## 2 Results and Discussion

### 2.1 Synthesis and Characterization of TCBQ/iLD‐ER NPs

**FIGURE 1:**
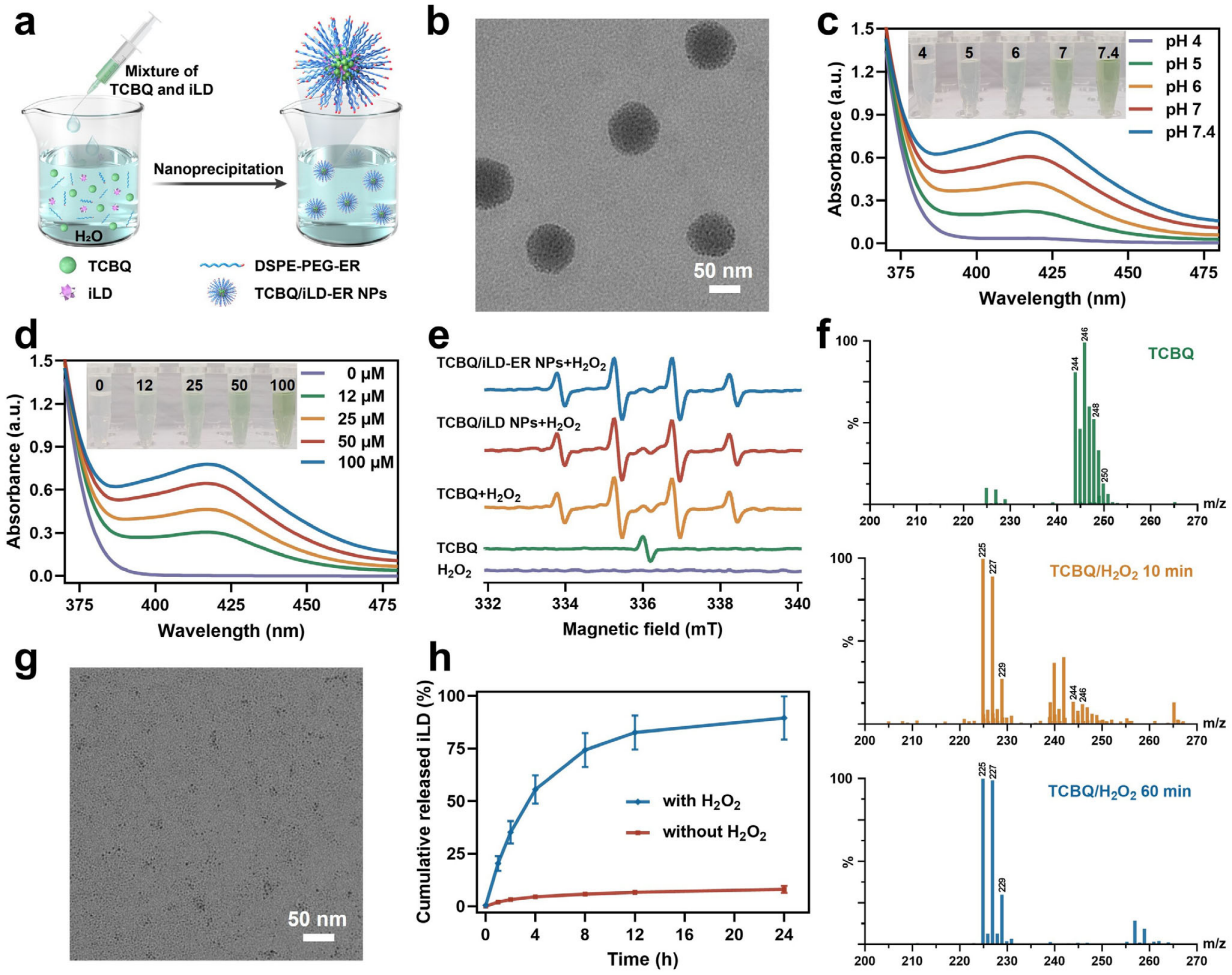
Characterization of TCBQ/iLD‐ER NPs. (a) Schematic diagram for the preparation of TCBQ/iLD‐ER NPs. (b) TEM image of TCBQ/iLD‐ER NPs. (c) UV‐Vis absorption spectra and photograph (inset) of ABTS after exposure to TCBQ/iLD‐ER NPs plus H_2_O_2_ at different pH. (d) UV‐Vis absorption spectra and photograph (inset) of ABTS after incubation with TCBQ/iLD‐ER NPs plus different concentrations of H_2_O_2_ at pH 7.4. (e) ESR spectra of •OH trapped by DMPO in TCBQ/iLD‐ER NPs + H_2_O_2_ system at pH 7.4. (f) ESI‐negative‐Q‐TOF‐MS spectra of TCBQ after incubation with H_2_O_2_ at pH 7.4 for different periods of time. (g) TEM image of TCBQ/iLD‐ER NPs after incubation with H_2_O_2_ at pH 7.4 for 6 h. (h) Cumulative iLD release from TCBQ/iLD‐ER NPs in the presence or absence of H_2_O_2_ at pH 7.4. n = 3. Data are presented as mean ± SD.

ER‐targeting moiety‐modified 1,2‐distearoyl‐sn‐glycero‐3‐phosphoethanolamine‐N‐[(polyethylene glycol)‐5000] (DSPE‐PEG‐ER) was acquired through an amido bond formation between N‐tosylethylenediamine and 1,2‐distearoyl‐sn‐glycero‐3‐phosphoethanolamine‐N‐[carboxy(polyethylene glycol)‐5000] (DSPE‐PEG‐COOH) (Figure S1). The resulting amphiphilic polymer DSPE‐PEG‐ER was characterized by ^1^H/^13^C nuclear magnetic resonance (NMR) spectroscopy (Figures S2 and S3). Additionally, zeta potential analysis showed that DSPE‐PEG‐COOH possessed a negative charge, while DSPE‐PEG‐ER had a near‐neutral charge (Figure S4), also suggesting that the carboxyl groups in DSPE‐PEG‐COOH were modified with ER‐targeting N‐tosylethylenediamine. Next, hydrophobic TCBQ and iLD were encapsulated into DSPE‐PEG‐ER via a nanoprecipitation approach to acquire ER‐targeted TCBQ/iLD‐ER NPs (Figure 1a). For comparison purposes, non‐ER‐targeted TCBQ/iLD NPs were synthesized using DSPE‐PEG‐COOH as an encapsulation matrix following the same procedure. The transmission electron microscopy (TEM) image displayed that the acquired TCBQ/iLD‐ER NPs possessed a monodisperse spherical morphology with an average diameter of around 80 nm (Figure 1b; Figure S5). The average hydrodynamic diameter measured by dynamic light scattering (DLS) was approximately 100 nm, which was slightly larger than that recorded by TEM (Figures S6 and S7).

Noteworthily, as a byproduct of protein folding in the ER lumen, the ER H_2_O_2_ maintains at a relatively high level [27, 28, 29, 30]. Thus, the feasibility of utilizing ER‐localized TCBQ/iLD‐ER NPs catalyzes the production of •OH from ER H_2_O_2_ was verified under the ER‐mimicking microenvironment of physiological pH. First, for confirming the chemodynamic performance of TCBQ/iLD‐ER NPs under organelle‐relevant pH conditions, the generation of free radicals was monitored by utilizing 2,2’‐azino‐bis(3‐ethylbenzothiazoline‐6‐sulfonic acid) diammonium salt (ABTS) that could be oxidized by ROS to oxidized ABTS (ox‐ABTS) with a signature absorption peak at about 420 nm. As displayed in Figure 1c,d, TCBQ/iLD‐ER NPs plus H_2_O_2_ led to a dramatic ABTS absorbance increase at 420 nm after 5 min of reaction at physiological pH 7.4, and minimal ABTS absorbance was detected under pH 4, indicating the potential of TCBQ/iLD‐ER NPs as a CDT agent capable of efficiently generating ROS under physiological pH conditions. Additionally, to identify the species of ROS produced in the TCBQ/iLD‐ER NPs + H_2_O_2_ system, 5,5‐dimethyl‐1‐pyrroline N‐oxide (DMPO) was selected as the spin‐trapping reagent. As shown in the electron spin resonance (ESR) spectra, characteristic spin signals with intensity ratio of 1:2:2:1 were monitored in TCBQ + H_2_O_2_, TCBQ/iLD NPs + H_2_O_2_, and TCBQ/iLD‐ER NPs + H_2_O_2_ groups (Figure 1e), manifesting the formation of DMPO‐•OH adduct, which could be attributed to TCBQ‐triggered conversion of H_2_O_2_ into •OH. Next, the mechanism of the reaction of TCBQ with H_2_O_2_ was investigated by electrospray ionization quadrupole time‐of‐flight mass spectrometry (ESI‐Q‐TOF‐MS). The ESI‐Q‐TOF‐MS analysis showed that the terminal product was water‐soluble TrCBQ‐O^−^ (Figure 1f), which contributes to the degradation of TCBQ/iLD‐ER NPs. As presented in the mechanism diagram (Figure S8), a nucleophilic reaction between TCBQ and H_2_O_2_ to form a quinone‐hydroperoxide reaction intermediate trichlorohydroperoxyl‐1,4‐benzoquinone (TrCBQ‐OOH) that could further decompose homolytically to generate highly cytotoxic •OH and trichlorohydroxy‐1,4‐benzoquinone radical (TrCBQ‐O•), TrCBQ‐O• may acquire an electron to form the ionic form of TrCBQ‐O^−^ [47, 48]. The H_2_O_2_‐triggered TCBQ/iLD‐ER NPs degradation was further verified after incubation with H_2_O_2_. As anticipated, TEM image showed that apparent disassembly of TCBQ/iLD‐ER NPs occurred after exposure to H_2_O_2_ at pH 7.4 (Figure 1g), and the hydrodynamic diameter also decreased with incubation time (Figure S9). Furthermore, sustained release of iLD from TCBQ/iLD‐ER NPs was detected in the presence of H_2_O_2_, whereas no distinct iLD release was observed in the absence of H_2_O_2_ (Figure 1h), corroborating the efficient iLD release triggered by H_2_O_2_. These results demonstrated the ability of TCBQ/iLD‐ER NPs as a CDT agent to generate •OH at physiological pH 7.4 and the H_2_O_2_‐responsive degradation of TCBQ/iLD‐ER NPs to release iLD as an LDs formation inhibitor.

### 2.2 In Vitro Chemodynamic Efficacy of TCBQ/iLD‐ER NPs

**FIGURE 2:**
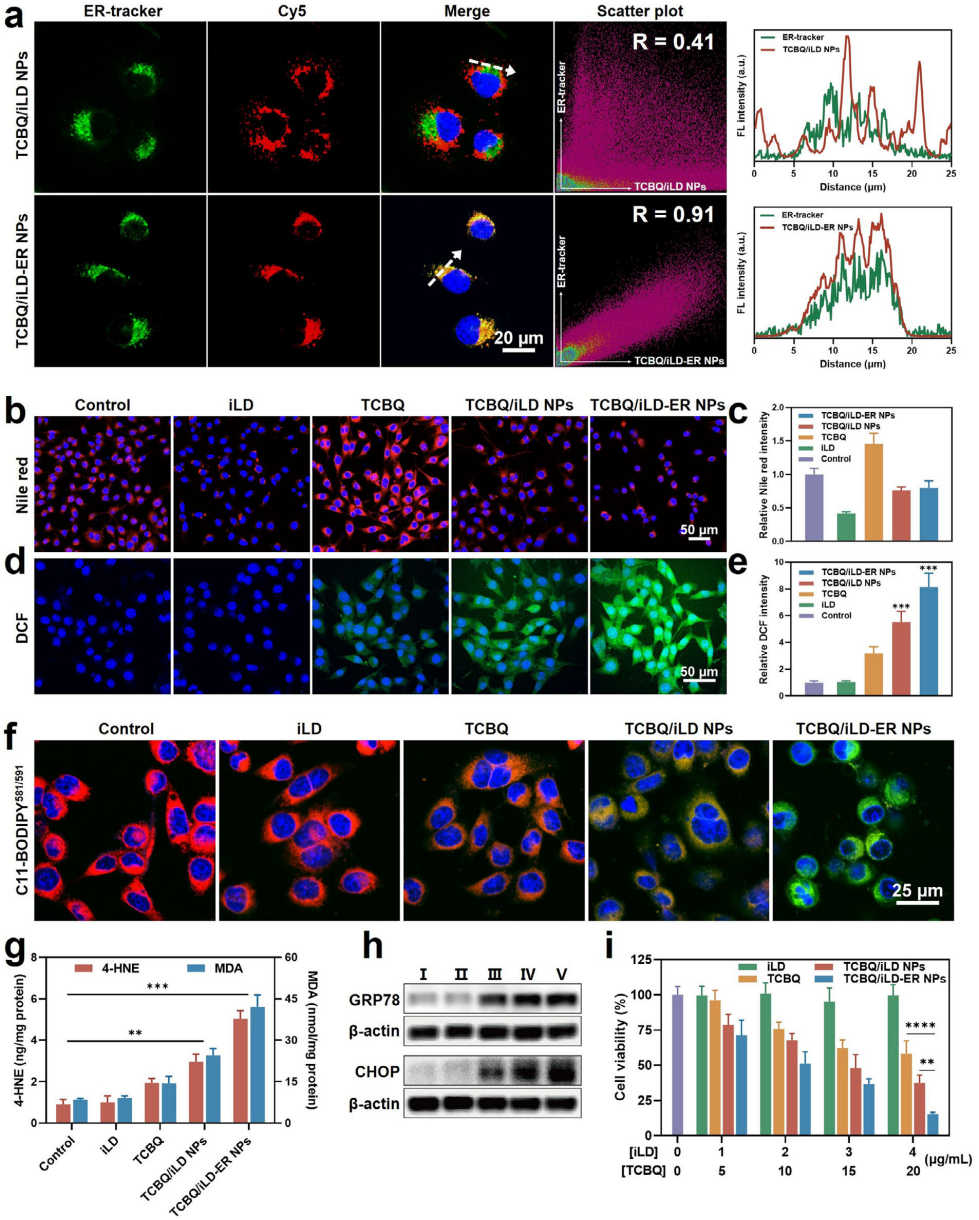
CDT performance of TCBQ/iLD‐ER NPs in vitro. (a) CLSM images of 4T1 cells incubated with Cy5‐labeled TCBQ/iLD NPs or TCBQ/iLD‐ER NPs (red channel) and ER‐tracker (green channel). The corresponding Pearson's correlation coefficients (R) were calculated. Fluorescence intensity profiles were along the corresponding white dashed arrows (25 µm). (b) CLSM images and (c) corresponding quantification of LDs levels in 4T1 cells by Nile red staining after incubation with different formulations for 12 h. Red color denotes Nile red‐stained LDs. n = 3. (d) CLSM images and (e) corresponding quantification of ROS levels in 4T1 cells by DCFH‐DA staining after incubation with different formulations for 4 h. n = 3. (f) C11‐BODIPY^581/591^ staining of 4T1 cells after incubation with different formulations for 6 h. (g) 4‐HNE and MDA content of 4T1 cells after 24 h of incubation with different formulations. n = 3. (h) Western blot analysis of GRP78 or CHOP expression in 4T1 cells after exposure to different formulations for 24 h. I: control, II: iLD, III: TCBQ, IV: TCBQ/iLD NPs, V: TCBQ/iLD‐ER NPs. (i) Cell viability of 4T1 cells after 24 h of incubation with diverse concentrations of iLD, TCBQ, TCBQ/iLD NPs, or TCBQ/iLD‐ER NPs. n = 3. Data are presented as mean ± SD. **p < 0.01, ***p < 0.001, ****p < 0.0001.

Considering for their potent ROS production capacity under physiological pH 7.4, the chemodynamic cytotoxicity of TCBQ/iLD‐ER NPs was tested. Before evaluating the CDT efficacy in vitro, 4T1 cells were treated with cyanine 5 (Cy5)‐labeled TCBQ/iLD‐ER NPs to investigate the cellular internalization of TCBQ/iLD‐ER NPs. As shown in the confocal laser scanning microscopy (CLSM) images, the fluorescence intensity within the cells increased with incubation time (Figure S10), demonstrating that TCBQ/iLD‐ER NPs can be effectively ingested by cancer cells. The subcellular localization of TCBQ/iLD‐ER NPs was also assessed by CLSM. In contrast to TCBQ/iLD NPs, TCBQ/iLD‐ER NPs preferentially accumulated in the ER which possessed high levels of H_2_O_2_ for triggering •OH production, as corroborated by a high co‐localization coefficient of 0.91 after co‐staining with commercial dye ER‐tracker green (Figure 2a), revealing that TCBQ/iLD‐ER NPs possessed superior capacity for selectively targeting the ER. Conversely, CLSM images displayed non‐significant differences among TCBQ/iLD NPs‐ and TCBQ/iLD‐ER NPs‐treated cells in mitochondrial co‐localization study (Figure S11).

LDs are dynamic organelles that serve vital roles in cellular metabolism [39]. ER stress could stimulate the formation of numerous LDs from the ER membrane [37, 38]. Importantly, increasing findings indicate that LDs function as buffers to prevent lipotoxic damage to intracellular organelles, enabling LDs to be a key mediator of tumor resistance to CDT and immunotherapy [49, 50]. LDs not only protect PUFAs from harmful peroxidation but also recruit CRT and thus block the transfer of CRT, thereby concurrently limiting both the chemodynamic cytotoxicity and immunity‐activating effect of CDT agents. On this account, the impact of different formulations on the sprout of LDs was assessed by Nile red staining. It can be observed that the amount of intracellular LDs declined dramatically upon iLD treatment, while TCBQ treatment obviously boosted LDs formation. Intriguingly, TCBQ‐induced increase in LDs content was significantly attenuated by concurrent treatment with iLD, as evidenced by the diminished LDs content in TCBQ/iLD NPs‐ or TCBQ/iLD‐ER NPs‐treated group (Figure 2b,c; Figure S12), implying that iLD exactly inhibited LDs generation triggered by TCBQ stimulation, which would be beneficial to enhance ER stress by disrupting the ER self‐protection mechanism. Next, the intracellular ROS level was evaluated by 2’,7’‐dichlorodihydrofluorescein diacetate (DCFH‐DA) assay, in which deacetylated form of DCFH‐DA could be oxidized by ROS to 2’,7’‐dichlorofluorescein (DCF) emitting green fluorescence. By comparing the DCF fluorescence intensity of CLSM images, it is obvious that TCBQ/iLD‐ER NPs‐treated cancer cells had a higher intracellular ROS level than TCBQ‐ and TCBQ/iLD NPs‐treated groups (Figure 2d,e). Concurrently, TCBQ/iLD‐ER NPs‐treated cells displayed the highest •OH production compared to TCBQ/iLD NPs (Figure S13), which elucidated that TCBQ/iLD‐ER NPs‐based CDT agents could exploit high levels of ER H_2_O_2_ to result in more extensive •OH generation. Then, the extent of cellular LPO resulting from •OH generated by TCBQ/iLD‐ER NPs was analyzed by ratiometric fluorescent probe C11‐BODIPY^581/591^. As can be seen in Figure 2f and Figure S14 (Supporting Information), in contrast to control cells with bright red fluorescence but minimal green fluorescence, 4T1 cells treated with TCBQ/iLD‐ER NPs exhibited markedly diminished red fluorescence and enhanced green fluorescence, indicating an elevated degree of LPO after exposure to the ER‐localized CDT nanoagents, which was positively correlated with intracellular ROS levels. Furthermore, LPO toxic byproducts including 4‑HNE and malondialdehyde (MDA) were significantly increased in cancer cells treated with TCBQ/iLD‐ER NPs as well (Figure 2g; Figure S15), which is beneficial for ICD induction due to the fact that 4‐HNE can directly modify ER proteins to interfere with protein folding and thereby initiate ER stress. High levels of LPO and LPO byproducts observed in TCBQ/iLD‐ER NPs‐treated cancer cells could be attributed to the combination effect of ROS generation and the inhibition of LDs formation. Notably, ER stress upregulates the levels of glucose‐regulated protein 78 (GRP78) and C/EBP homologous protein (CHOP) [33, 35]. As shown in Western blot analysis, the TCBQ/iLD‐ER NPs‐treated cells exhibited higher GRP78 or CHOP expression than that of TCBQ/iLD NPs group (Figure 2h; Figure S16), indicating that ER‐localized TCBQ/iLD‐ER NPs could more effectively induce ER stress. In addition, the chemodynamic toxicity of TCBQ/iLD‐ER NPs against tumor cells was quantitatively assessed by the cell counting kit‐8 (CCK‐8) analysis. As predicted, the viability of TCBQ/iLD‐ER NPs‐treated 4T1 cells was distinctly lower than that of TCBQ/iLD NPs‐treated cells, because the relatively high levels of H_2_O_2_ present in the ER increased chemodynamic •OH production and thereby potentiated CDT efficacy (Figure 2i; Figure S17). Similarly, the live/dead assay and apoptosis analysis revealed that TCBQ/iLD‐ER NPs caused more efficient cancer cell death than iLD, TCBQ, or TCBQ/iLD NPs (Figures S18 and S19), further demonstrating the excellent chemodynamic therapeutic effect of TCBQ/iLD‐ER NPs. Thus, the ER‐localized TCBQ/iLD‐ER NPs could fully utilize high levels of ER H_2_O_2_ for sufficient chemodynamic •OH generation, which could not only enhance chemodynamic toxicity against cancer cells but also in situ induce ER stress.

### 2.3 ICD‐Inducing Effect of TCBQ/iLD‐ER NPs In Vitro

**FIGURE 3:**
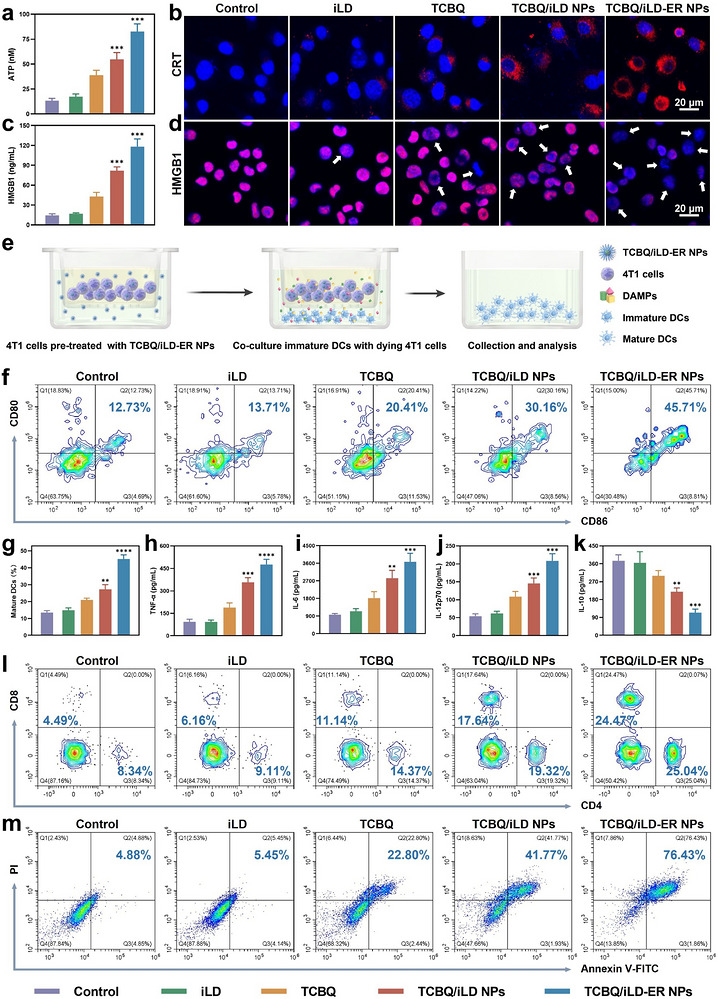
Chemodynamically generated •OH‐induced ICD and immune responses by TCBQ/iLD‐ER NPs in vitro. (a) ATP release from 4T1 cells after incubation with different formulations for 24 h. n = 3. (b) CLSM images displaying CRT exposure on the surface of 4T1 cells after incubation with different formulations for 24 h. (c) ELISA assay of extracellular HMGB1 content and (d) CLSM images displaying intracellular HMGB1 of 4T1 cells after incubation with different formulations for 24 h, altogether demonstrating marked efflux of HMGB1. n = 3. (e) Schematic illustration of the co‐incubation system of 4T1 cells and immature DCs in vitro. (f) Surface expression of CD80 and CD86 on DCs after co‐culture with different formulation‐pretreated 4T1 cells quantitatively analyzed by FCM (gating on CD11c^+^) and (g) corresponding quantification histogram. n = 3. ELISA analysis of the secretion of (h) TNF‐α, (i) IL‐6, (j) IL‐12p70, and (k) IL‐10 from DCs co‐cultured with different formulation‐pretreated 4T1 cells. n = 3. (l) Surface expression of CD8 or CD4 on T cells after co‐culture with pre‐educated DCs quantitatively analyzed by FCM (gating on CD3^+^). (m) FCM analysis of 4T1 cells apoptosis after co‐culture with pre‐activated T cells. Data are presented as mean ± SD. **p < 0.01, ***p < 0.001, ****p < 0.0001.

In addition to the direct tumoricidal effect based on their chemodynamic cytotoxicity, the ability of TCBQ/iLD‐ER NPs to initiate ER stress‐induced ICD was also validated in vitro. The induction of cancer cell ICD is an effective therapeutic approach to enable the organism to acquire systemic anti‐tumor immunity [51, 52]. ER stress‐mediated ICD in tumor cells leads to the massive release of DAMPs, including ATP, CRT, and HMGB1 [11]. As for ATP, dying cells release extracellularly, deliver “find‐me” signals to DCs precursor cells and macrophages, driving the recruitment of myeloid cells toward active ICD regions. The exposure of CRT to the plasma membrane of dying cells liberates “eat‐me” signals, encourages DCs to phagocytose tumor cells, and stimulates the maturation of DCs. HMGB1, the cytokine associated with inflammatory response, facilitates the steady binding of DCs to dying tumor cells, which serves an essential part in the process of inducing the maturation of DCs. On this account, DAMPs release was comprehensively analyzed to validate the ability of ER‐localized CDT nanoagents to induce ICD. As a prelude, ATP release into extracellular supernatant was quantified by a bioluminescent assay kit, which is known as one of the hallmarks of ICD. As displayed in Figure 3a, TCBQ/iLD‐ER NPs‐treated cells exhibited elevated release of ATP compared to the control group. Next, the exposure of CRT on 4T1 cells was monitored employing CLSM. In contrast to 4T1 cells treated with iLD or TCBQ alone, intense red fluorescence was detected in TCBQ/iLD‐ER NPs‐treated group (Figure 3b; Figure S20), elucidating obvious CRT expression on the cell surface. Noteworthily, the iLD‐mediated inhibition of LDs formation contributes to the robust CRT exposure through reducing the recruitment of CRT by LDs, as evidenced by co‐localization analysis (Figure S21). Besides ATP secretion and CRT translocation, the HMGB1 migrates from the nucleus to cytoplasm and then releases into extracellular as another indicator of ICD induction. The HMGB1 level in TCBQ/iLD‐ER NPs‐treated cancer cells was investigated by the detection kit and immunofluorescence assay. As depicted in Figure 3c,d and Figure S22 (Supporting Information), nuclear HMGB1 efflux was observed in TCBQ/iLD‐ER NPs‐treated group compared to the non‐ER‐targeted TCBQ/iLD NPs‐treated cells, indicating that ER targeting further improved ICD efficiency due to the relatively higher level of ER H_2_O_2_ for chemodynamic •OH generation. Evidently, TCBQ/iLD‐ER NPs‐treated cells could effectively evoke the release of ICD‐related DAMPs, including ATP secretion, CRT exposure, and HMGB1 efflux. Altogether, these results confirmed that ER‐localized TCBQ/iLD‐ER NPs can elicit severe ER stress for priming downstream enhanced ICD by the combination of intensive •OH production with LDs inhibition.

During the ICD process, the released DAMPs can be identified by pattern recognition receptors (PRRs) located on antigen‐presenting cells (APCs) surfaces, allowing the activation of APCs [53, 54]. DCs are one of the most vital APCs, and mature DCs with high expression of CD80 and CD86 markers promote the activation of naïve T cells to activate innate and adaptive immunity. Thereby, the maturation level of DCs is positively associated with the grade of T cells activation. To evaluate the maturity of DCs mediated by TCBQ/iLD‐ER NPs‐triggered ICD in vitro, bone‐marrow‐derived DCs were extracted and then co‐cultured with different formulation‐pretreated 4T1 cells, followed by flow cytometry (FCM) analysis (Figure 3e). The costimulatory molecules (CD80 and CD86) are markers of mature DCs, and TCBQ/iLD‐ER NPs‐pretreated 4T1 cells dramatically increased the proportion of mature DCs (CD80^+^CD86^+^ DCs) as compared to the control group (Figure 3f,g), which implied that DAMPs released from TCBQ/iLD‐ER NPs‐pretreated cancerous cells potently induced DCs maturation, permitting ICD‐mediated immune activation. Significantly, several pro‐inflammatory cytokines secreted from mature DCs can stimulate T cell‐mediated immune responses. Thus, the enzyme‐linked immunosorbent assay (ELISA) was performed to assess the secretion of cytokines related to DCs activation. After incubation with TCBQ/iLD‐ER NPs‐pretreated 4T1 cells, the secretion of pro‐inflammatory cytokines by mature DCs was markedly elevated, such as tumor necrosis factor α (TNF‐α), interleukin 6 (IL‐6), and interleukin 12p70 (IL‐12p70), which could contribute to the promotion of T cells proliferation, while the secretion of the immune‐suppressor cytokine interleukin 10 (IL‐10) was diminished (Figure 3h–k). Next, to assess the capacity of DCs to activate T cells, spleen‐derived T cells were co‐cultured with pre‐educated DCs and analyzed by FCM. Compared with the control group, the CD3^+^CD8^+^ T cells (cytotoxic T lymphocytes) and CD3^+^CD4^+^ T cells (helper T lymphocytes) in TCBQ/iLD‐ER NPs group increased remarkably (Figure 3l), indicating that tumor antigen‐pre‐educated DCs can effectively prime T cells. Subsequently, following co‐culture of pre‐activated T cells with 4T1 cells, a high rate of apoptosis was observed in the TCBQ/iLD‐ER NPs group, whereas a lower apoptosis rate was detected in the control group (Figure 3m), implying that activated T cells possess tumor‐specific killing ability. The above results supported the feasibility of using TCBQ/iLD‐ER NPs to trigger antitumor immunity. Accordingly, TCBQ/iLD‐ER NPs, given their ability to increase the immunogenicity of cancer cells, could potentially be used as chemodynamic immunotherapeutic agents to inhibit tumor growth and metastasis.

### 2.4 Tumor Inhibition Ability of TCBQ/iLD‐ER NPs In Vivo

**FIGURE 4:**
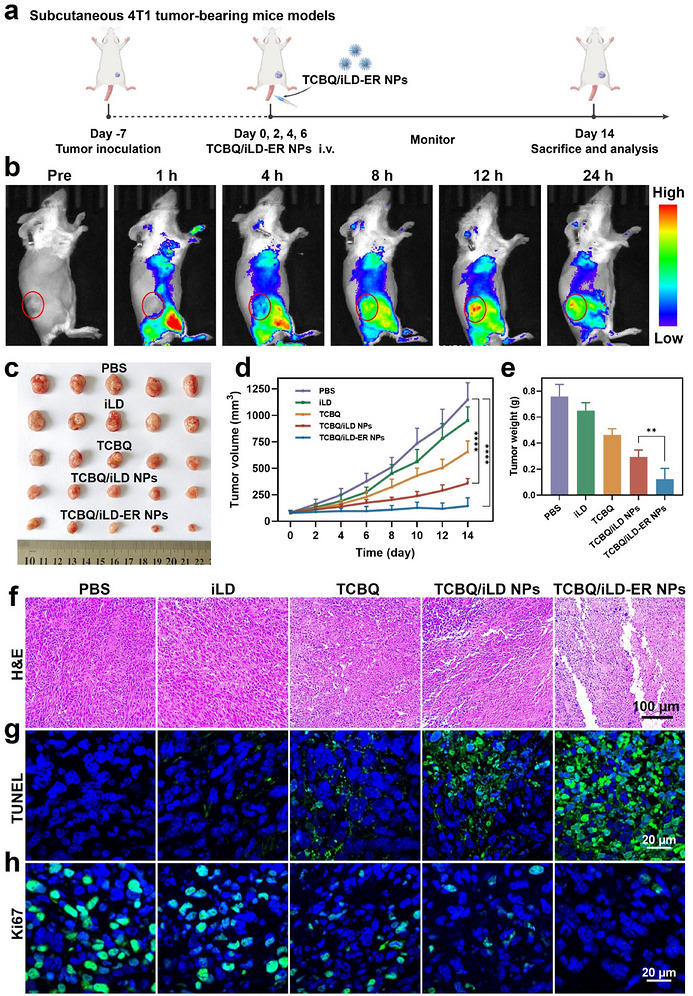
Tumor accumulation and anti‐tumor efficacy of TCBQ/iLD‐ER NPs after i.v. injection. (a) Schematic illustration of the therapeutic protocol for 4T1 tumor‐bearing mice in vivo. (b) In vivo fluorescence images of 4T1 tumor‐bearing mice after i.v. administration of Cy5‐labeled TCBQ/iLD‐ER NPs at various time points. The red circles highlight the tumor region. (c) Photograph of tumors harvested from mice on the 14th day post‐injection with different formulations. n = 5. (d) Tumor growth curves of 4T1 tumor‐bearing mice post‐injection with different formulations. n = 5. (e) The weight of tumors harvested from mice on the 14th day post‐injection with different formulations. n = 5. (f) H&E, (g) TUNEL, and (h) Ki67 staining of tumor slices from various groups. Data are presented as mean ± SD. **p < 0.01, ****p < 0.0001.

Inspired by the favorable anticancer performance of TCBQ/iLD‐ER NPs in vitro, we proceeded to investigate the antitumor effect in vivo (Figure 4a). First, TCBQ/iLD‐ER NPs exhibited negligible hemolysis effect even at high concentrations (Figure S23), indicating that they could be safely employed in in vivo experiments. Next, Cy5‐labeled TCBQ/iLD‐ER NPs were injected intravenously (i.v.) and their biodistribution was monitored using an in vivo imaging system (IVIS). Fluorescence images exhibited that the fluorescence signal at the tumor region was gradually enhanced and achieved the maximum value at 12 h (Figure 4b). The ex vivo organ fluorescence images displayed still high accumulation of TCBQ/iLD‐ER NPs in the tumor region at 24 h post‐injection (Figure S24), affirming efficient tumor accumulation of TCBQ/iLD‐ER NPs. To explore the tumor suppression effect of TCBQ/iLD‐ER NPs in vivo, 4T1 tumor‐bearing mice were randomly allocated into five groups, and i.v. administered with different formulations every other day for 4 doses. As presented in the excised tumor and tumor growth curves, tumor growth was remarkably restrained in mice treated with TCBQ/iLD‐ER NPs compared to phosphate buffered saline (PBS)‐treated mice (Figure 4c–e; Figure S25), which was ascribed to the prominent chemodynamic effect of TCBQ/iLD‐ER NPs against tumor cells. By contrast, non‐ER‐targeted TCBQ/iLD NPs were inferior to TCBQ/iLD‐ER NPs in anti‐tumor efficacy. In addition, hematoxylin and eosin (H&E) staining and terminal deoxynucleotidyl transferase dUTP nick end labeling (TUNEL) immunofluorescent staining assays revealed the TCBQ/iLD‐ER NPs‐caused severely damage in tumor tissues (Figure 4f,g), further providing compelling evidence of the excellent anti‐tumor effect of TCBQ/iLD‐ER NPs. Moreover, proliferation marker Ki67 immunofluorescent staining assay was also performed to confirm the inhibition of tumor proliferation by TCBQ/iLD‐ER NPs. As observed in Figure 4h, the expression of Ki67 was notably decreased in tumors from TCBQ/iLD‐ER NPs‐treated mice. Meanwhile, the CD8 and CD4 immunofluorescent staining analysis manifested that the mice treated with TCBQ/iLD‐ER NPs exhibited higher proliferation of T cells at the tumor region compared to the non‐ER‐targeted TCBQ/iLD NPs group (Figure S26), which is in favour of evoking anti‐tumor immune responses. Additionally, neither obvious fluctuations in body weights or blood biochemical indexes nor detectable histological damage in the major organs were observed in TCBQ/iLD‐ER NPs‐treated mice (Figures S27–S29), suggesting the biosafety of TCBQ/iLD‐ER NPs in vivo. Taken together, these encouraging findings supported that TCBQ/iLD‐ER NPs are prospective CDT nanoagents for tumor treatment.

### 2.5 Abscopal Effects of TCBQ/iLD‐ER NPs In Vivo

**FIGURE 5:**
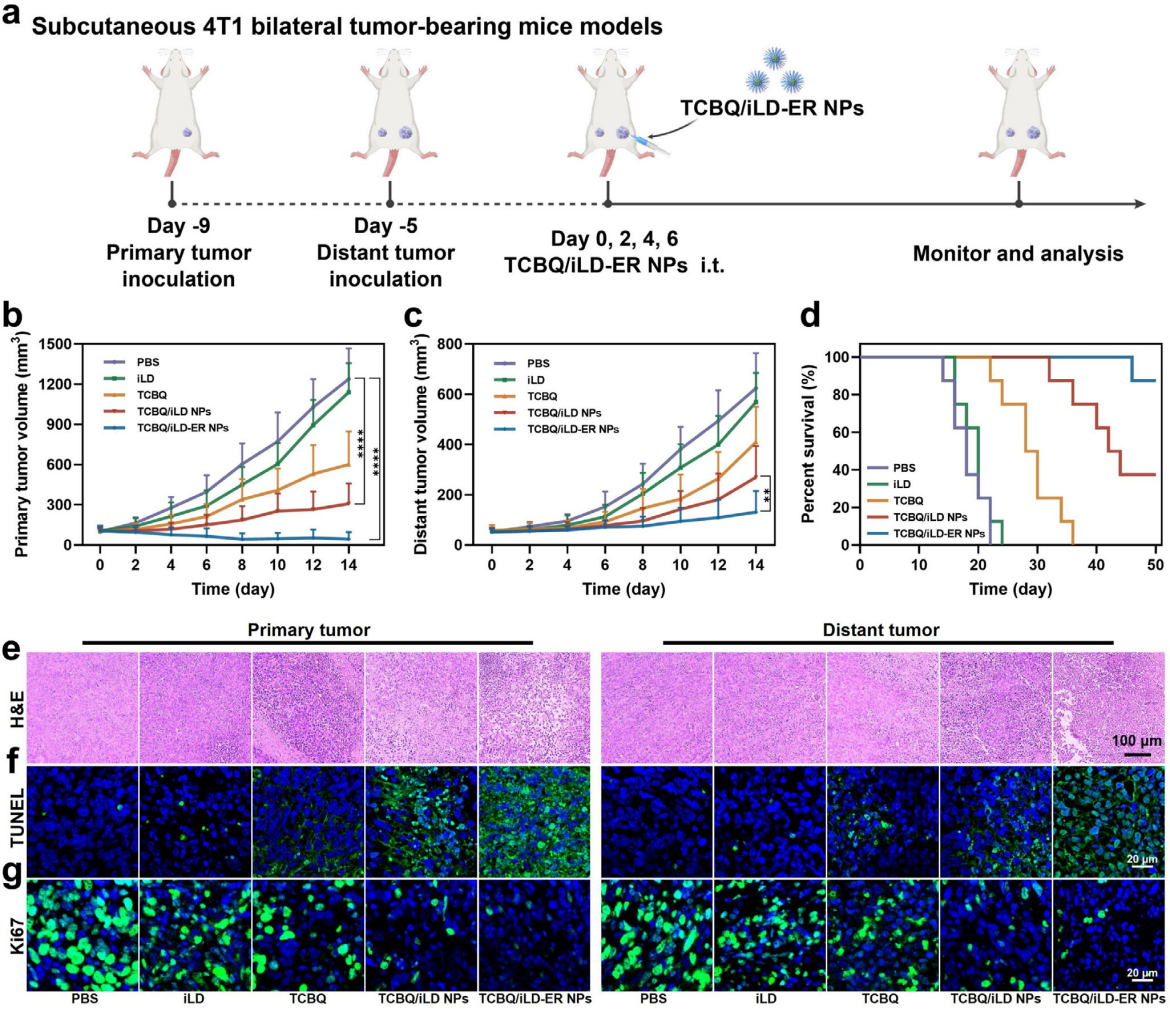
Abscopal effects of TCBQ/iLD‐ER NPs in vivo. (a) Schematic illustration of the therapeutic protocol for 4T1 bilateral tumor‐bearing mice in vivo. (b) Primary and (c) distant tumor growth curves of 4T1 bilateral tumor‐bearing mice post‐injection with different formulations. n = 8. (d) Survival curves of mice post‐injection with different formulations. n = 8. (e) H&E, (f) TUNEL, and (g) Ki67 staining of tumor slices from various groups. Data are presented as mean ± SD. **p < 0.01, ****p < 0.0001.

Based on their favorable antitumor properties after i.v. administration, we further investigated whether TCBQ/iLD‐ER NPs could elicit tumor immunogenicity to inhibit metastatic tumors in vivo. Therefore, 4T1 bilateral tumor models were established and tumor treatment was commenced when the right/primary tumor reached approximately 100 mm^3^ and the left/distant tumor reached around 50 mm^3^ (Figure 5a). To begin with, 4T1 bilateral tumor‐bearing mice were randomly allocated into five groups and intratumorally (i.t.) administered with different formulations at the primary tumor sites. As can be observed, the TCBQ/iLD‐ER NPs‐treated mice exhibited remarkable bilateral tumors suppression owing to the superior chemodynamic immunotherapeutic effect of TCBQ/iLD‐ER NPs (Figure 5b,c; Figure S30). Furthermore, mice treated with TCBQ/iLD‐ER NPs displayed considerably prolonged life span compared to PBS‐treated group (Figure 5d). Additionally, the histological study elucidated that more serious tumor cells damage was found in bilateral tumor tissues of TCBQ/iLD‐ER NPs‐treated mice than in that of other groups, as evidenced by the H&E staining (Figure 5e). The abscopal antitumor effects of TCBQ/iLD‐ER NPs were also confirmed by immunofluorescence staining. TUNEL immunofluorescence staining revealed the severest apoptosis in distant tumor tissues after TCBQ/iLD‐ER NPs treatment, and Ki67 immunofluorescence staining affirmed remarkable inhibition of distant tumor proliferation (Figure 5f,g). Moreover, both primary and distant tumors in mice injected with TCBQ/iLD‐ER NPs showed pronounced proliferation of T cells by CD8 and CD4 immunofluorescence analysis (Figure S31), which was consistent with the findings of tumor growth suppression. Also, there were no abnormalities in body weight loss or organ sections in all treated mice (Figures S32 and S33). The results displayed the abscopal antitumor efficacy of TCBQ/iLD‐ER NPs to effectively suppress the growth of both primary and distant tumors.

### 2.6 Immunity Activation Ability of TCBQ/iLD‐ER NPs In Vivo

**FIGURE 6:**
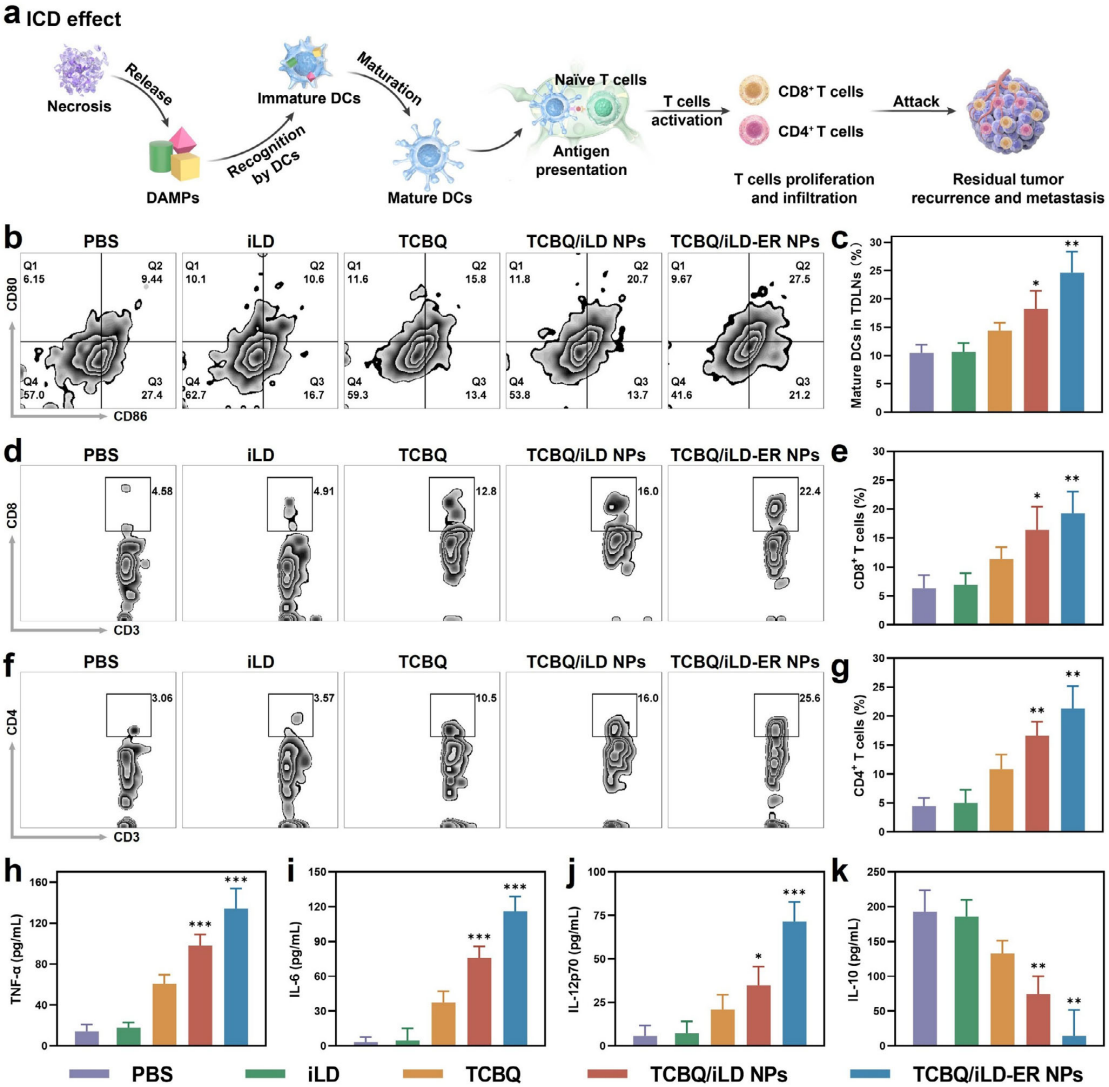
TCBQ/iLD‐ER NPs‐induced anti‐tumor immune responses in vivo. (a) Schematic illustration of DCs maturation and T cells proliferation triggered by TCBQ/iLD‐ER NPs‐mediated tumor ICD. (b) Surface expression of CD80 and CD86 on DCs within distant TDLNs from mice after treatment with different formulations quantitatively analyzed by FCM (gating on CD11c^+^) and (c) corresponding quantification histogram. n = 3. (d) Surface expression of CD8 on T cells within distant tumors from mice after treatment with different formulations quantitatively analyzed by FCM (gating on CD3^+^) and (e) corresponding quantification histogram. n = 3. (f) Surface expression of CD4 on T cells within distant tumors from mice after treatment with different formulations quantitatively analyzed by FCM (gating on CD3^+^) and (g) corresponding quantification histogram. n = 3. ELISA analysis of the secretion of (h) TNF‐α, (i) IL‐6, (j) IL‐12p70, and (k) IL‐10 in serum from mice after treatment with different formulations. n = 3. Data are presented as mean ± SD. *p < 0.05, **p < 0.01, ***p < 0.001.

Aiming to clarify the mechanism of abscopal effects induced by local injection of TCBQ/iLD‐ER NPs‐based chemodynamic immunotherapeutic agents, we evaluated immune cells in the tumors and spleens of treated mice, as well as the relevant immune cytokines in the serum (Figure 6a). At first, the FCM was performed to investigate the CD80/CD86 expression on DCs within tumor‐draining lymph nodes (TDLNs) collected from different groups, which is pivotal for ICD immune response. As compared to the control group, the proportion of CD80^+^CD86^+^ DCs in the TCBQ/iLD‐ER NPs‐treated group was obviously elevated (Figure 6b,c), demonstrating effective maturation of DCs. Then, the infiltration of CD3^+^CD8^+^ T cells and CD3^+^CD4^+^ T cells in distant tumors after different treatments was also examined to verify systemic immune response activation. Likewise, the proportions of CD3^+^CD8^+^ T cells and CD3^+^CD4^+^ T cells were increased in the distant tumors of TCBQ/iLD‐ER NPs‐treated mice (Figure 6d–g). These findings pointed out higher levels of infiltration of T lymphocytes within the distant tumors of TCBQ/iLD‐ER NPs‐treated mice, which contributed to anti‐tumor immunotherapy. Additionally, sustained antigenic stimulation commonly promotes T cell exhaustion (Tex, CD3^+^CD8^+^TIM‐3^+^). The TCBQ/iLD‐ER NPs‐treated mice exhibited the lowest proportion of Tex in the distant tumor compared to other groups (Figure S34), suggesting that TCBQ/iLD‐ER NPs can potently mitigate Tex and uphold cytotoxic T lymphocytes activity. Subsequently, the proportions of CD3^+^CD8^+^ T cells and CD3^+^CD4^+^ T cells, as well as effector memory T cells (Tem, CD3^+^CD8^+^CD44^+^CD62L^−^) and central memory T cells (Tcm, CD3^+^CD8^+^CD44^+^CD62L^+^), in the spleens were also examined. As can be seen in Figures S35 and S36 (Supporting Information), the percentages of CD3^+^CD8^+^ T cells, CD3^+^CD4^+^ T cells, and memory T cells in spleens harvested from TCBQ/iLD‐ER NPs‐treated group were distinctly higher than that from PBS‐treated control group, which indicated that the induction of tumor ICD by TCBQ/iLD‐ER NPs successfully evoked systemic anti‐tumor immune effects and conferred long‐lasting immunity. The FCM results demonstrated the activation of systemic anti‐tumor immune responses by local injection of TCBQ/iLD‐ER NPs. Next, various cytokines in the serum of mice after treatment with TCBQ/iLD‐ER NPs were analyzed with ELISA. Among them, the pro‐inflammatory cytokines, including TNF‐α, IL‐6, and IL‐12p70, were markedly upregulated in the serum, while the immune‐suppressor cytokine IL‐10 was obviously reduced (Figure 6h–k), implying that the recruited immune cells within tumor site produced abundant immune factors to initiate valid ICD. Taken together, these findings showed the great potential of TCBQ/iLD‐ER NPs in provoking anti‐tumor immune responses.

### 2.7 Tumor Metastasis Inhibition by TCBQ/iLD‐ER NPs In Vivo

**FIGURE 7:**
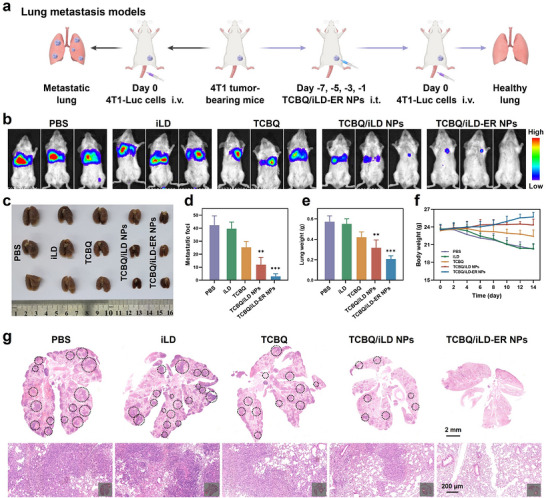
Anti‐metastasis efficacy of TCBQ/iLD‐ER NPs in vivo. (a) Schematic illustration of the therapeutic protocol for lung metastasis models. (b) Bioluminescence images of metastatic 4T1‐Luc cells in mice after different treatments. (c) Photograph of lungs harvested from mice after different treatments. (d) Quantifications of lung metastatic nodules. n = 3. (e) The weight of lungs from mice after treatment with different formulations. n = 3. (f) Body weight curves of 4T1 tumor‐bearing mice after treatment with different formulations. (g) Representative H&E‐stained images of lung at different groups (metastatic nodules were marked with black dashed circles). Data are presented as mean ± SD. **p < 0.01, ***p < 0.001.

Tumor metastasis is a major cause of death in cancer patients, and lung is the most prevalent area of metastasis [55]. In order to investigate the efficacy of the ICD inducer TCBQ/iLD‐ER NPs in combating tumor metastasis, we established lung metastasis models by i.v. injecting 4T1‐Luc cells into 4T1 tumor‐bearing mice after treatment with different formulations (Figure 7a). Bioluminescence imaging was conducted to examine 4T1‐Luc cells metastasis. As can be seen in Figure 7b, the bioluminescence intensities in chest areas of TCBQ/iLD‐ER NPs‐treated mice were significantly weaker than that of PBS‐treated group, indicating that tumor metastasis in tumor‐bearing mice was remarkably suppressed after TCBQ/iLD‐ER NPs treatment. Next, the lungs were isolated from different groups at the end of observation period, followed by further analysis. The lung tissues of control group exhibited obvious metastatic nodules, while the lungs of TCBQ/iLD‐ER NPs‐treated mice were smooth with no apparent metastatic nodules (Figure 7c,d), underscoring the capacity of TCBQ/iLD‐ER NPs to suppress lung metastasis. Moreover, the lung weight of TCBQ/iLD‐ER NPs‐treated mice was lower than that of PBS‐treated control mice (Figure 7e). Additionally, the body weight was also recorded to assess the health status of mice. As shown in Figure 7f, the body weight of TCBQ/iLD‐ER NPs‐treated mice increased steadily, while the control group kept decreasing, suggesting that tumor metastasis led to poor physical condition of PBS‐treated mice. H&E‐stained images also showed noticeable lung metastasis in the control group, while no obvious metastatic nodules were observed in the lung tissues of TCBQ/iLD‐ER NPs‐treated mice (Figure 7g). These results displayed that the systemic antitumor immune response activated by the chemodynamic immunotherapeutic effect of TCBQ/iLD‐ER NPs effectively inhibited tumor metastasis.

## 3 Conclusions

In conclusion, we have developed an ER‐targeting CDT nanoagent capable of efficiently generating •OH under physiological pH conditions to enhance chemodynamic immunotherapy by inhibiting the formation of intracellular LDs. On the one hand, the nanoagent employs iLD to break the dual restrictions of LDs on chemodynamic cytotoxicity and immunity‐activating effect of CDT agents. On the other hand, unlike classical Fenton chemistry‐based CDT systems, TCBQ/iLD‐ER NPs bypass the acidic pH constraint inherent, allowing efficient production of cytotoxic •OH from H_2_O_2_ inside the ER lumen with physiological pH. After endocytosis by cancerous cells, TCBQ/iLD‐ER NPs preferentially accumulated in the ER, where TCBQ could react with high levels of ER H_2_O_2_ to yield abundant •OH accompanied by the degradation of TCBQ/iLD‐ER NPs to release iLD. Interestingly, LPO of PUFAs induced by the chemodynamically generated •OH not only caused cancer cell death, but also produced 4‐HNE as a byproduct to provoke ER stress‐mediated ICD. Furthermore, the released iLD inhibited the formation of LDs and thereby reduced the sequestration of PUFAs as well as the recruitment of CRT by LDs, allowing TCBQ/iLD‐ER NPs to provide improved chemodynamic immunotherapeutic effects. After treatment with TCBQ/iLD‐ER NPs, both primary and distant tumors as well as cancer metastasis were significantly suppressed, prolonging the life span of tumor‐bearing mice. This study demonstrates the feasibility of using TCBQ/iLD‐ER NPs to intensify chemodynamic immunotherapy of cancer via inhibiting LDs formation and will open new avenues for promoting the antitumor efficacy of ROS‐based immunotherapies.

## Ethical Statement

Animal experiments were performed according to animal protocols approved by the Institutional Animal Care and Use Committee of Fuzhou University and Fujian Medical University (IACUC FJMU 2022‐0608).

## Statistical Analysis

All quantitative data were expressed as the mean ± standard deviation (SD). Data analyses were conducted using the GraphPad Prism 9 software. Statistical analyses were performed using the Student's two‐tailed t‐test. Unless otherwise specified, all comparisons were made relative to the control group. P values of < 0.05 were considered significant. *p < 0.05, **p < 0.01, ***p < 0.001, ****p < 0.0001.

## Conflicts of Interest

The authors declare no conflicts of interest.

## Supporting information




**Supporting File**: advs75626‐sup‐0001‐SuppMat.docx.

## Data Availability

The data that support the findings of this study are available from the corresponding author upon reasonable request.
