# Supplementary material for: Amplification of Endoplasmic Reticulum Stress via Inhibiting Lipid Droplet Formation to Enhance Chemodynamic Immunotherapy
Source: Adv Sci (Weinh). 2026 May 10;13(44):e75626. doi: 10.1002/advs.75626 (PMC13335917; doi:10.1002/advs.75626)
Supplement: Supplementary file 1 [file ADVS-13-e75626-s001.docx]

Supporting Information

**Amplification of Endoplasmic Reticulum Stress via** **Inhibiting Lipid Droplet Formation to Enhance Chemodynamic Immunotherapy**

*Huilan Cai,*^+^ *Shaoru Zhuang,*^+^ *Yang Zhu,*^+^ *Rujiang Ao, Meili Yu, Tingting Cui, Jun Wang, Xuegang Niu, Hongwei Huang, Shanshan Peng, Yu He,** *Lisen Lin,** *and Huanghao Yang**

Experimental Methods

**Chemicals and reagents**

N-tosylethylenediamine (97%), N-hydroxysuccinimide (NHS), N-(3-dimethylaminopropyl)-N’-ethylcarbodiimide hydrochloride (EDC), tetrachloro-1,4-benzoquinone (TCBQ, 99%), hydrogen peroxide (H_2_O_2_, 30%), 2,2’-azino-bis(3-ethylbenzothiazoline-6-sulfonic acid) diammonium salt (ABTS, ≥ 98%), 5,5-dimethyl-1-pyrroline N-oxide (DMPO), and Nile red (≥ 97%) were purchased from Sigma-Aldrich (St. Louis, USA). A-922500 was purchased from MedChemExpress (New Jersey, USA). 1,2-distearoyl-sn-glycero-3-phosphoethanolamine-N-[carboxy(polyethylene glycol)-5000] (DSPE-PEG-COOH) was purchased from Beijing Warwick Chemical Co., Ltd. (Beijing, China). Cyanine5-n-hydroxysuccinimide ester (Cy5-NHS) was purchased from Shanghai Yuanye Bio-Technology Co., Ltd. (Shanghai, China). ER-tracker green and C11-BODIPY^581/591^ were purchased from Thermo Fisher Scientific Inc. (Eugene, USA). Hoechst 33342, 2’,7’-dichlorodihydrofluorescein diacetate (DCFH-DA), lipid peroxidation (LPO) malondialdehyde (MDA) assay kit, annexin V-FITC/propidium iodide (PI) apoptosis detection kit, adenosine triphosphate (ATP) assay kit, and high mobility group box 1 (HMGB1) enzyme-linked immunosorbent assay (ELISA) kit were purchased from Beyotime Biotechnology (Shanghai, China). Mouse 4-hydroxynonenal (4-HNE) ELISA kit was purchased from Fine Biotech Co., Ltd. (Wuhan, China). Cell counting kit-8 (CCK-8) and calcein-AM/PI double staining kit were purchased from Dojindo Molecular Technologies, Inc. (Kumamoto, Japan). The ELISA kits for tumor necrosis factor α (TNF-α), interleukin 6 (IL-6), interleukin 12p70 (IL-12p70), and interleukin 10 (IL-10) were purchased from Multi Sciences (Hangzhou, China). Anti-glucose-regulated protein 78 (GRP78) rabbit monoclonal antibody (3177) and anti-C/EBP homologous protein (CHOP) rabbit monoclonal antibody (5554) for Western blot analysis were purchased from Cell Signaling Technology, Inc. (Danvers, USA). Anti-calreticulin (CRT) rabbit monoclonal antibody (ab92516), anti-HMGB1 rabbit monoclonal antibody (ab79823), goat anti-rabbit IgG H&L (Alexa Fluor^®^ 488) (ab150077), and goat anti-rabbit IgG H&L (Alexa Fluor^®^ 568) (ab175471) for confocal laser scanning microscope (CLSM) observation were purchased from Abcam Ltd. (Cambridge, UK). Anti-CD11c-FITC (Catalog: 117305, Clone: N418), anti-CD80-PE (Catalog: 104707, Clone: 16-10A1), anti-CD86-APC (Catalog: 105011, Clone: GL-1), anti-CD3ε-FITC (Catalog: 100305, Clone: 145-2C11), anti-CD8a-PE (Catalog: 100707, Clone: 53-6.7), anti-CD4-APC (Catalog: 100411, Clone: GK1.5), anti-CD44-APC (Catalog: 103011, Clone: IM7), anti-CD62L-APC/Cyanine7 (Catalog: 104427, Clone: MEL-14), and anti-TIM-3-APC (Catalog: 119705, Clone: RMT3-23) monoclonal antibodies for flow cytometry (FCM) analysis were purchased from BioLegend, Inc. (California, USA). All commercially available chemicals and reagents were used directly without further purification. Deionized (DI) water was acquired from the Milli-Q water purification systems (Millipore, Billerica, USA).

**Instruments**

Nuclear magnetic resonance (NMR) spectra were performed by 600MHz superconducting nuclear magnetic resonance spectrometer (AVANCE NEO 600M, Bruker). Transmission electron microscopy (TEM) images were acquired by HT7700 transmission electron microscope (Hitachi, Japan). Hydrodynamic diameters and zeta potentials were measured by Malvern zetasizer nano ZS (Malvern, UK). Ultraviolet-visible (UV-Vis) absorption spectra were analyzed by UH4150 spectrophotometer (Hitachi, Japan). The release of A-922500 (a lipid droplets (LDs) formation inhibitor, defined as iLD) from TCBQ/iLD-ER NPs was measured by ultra performance liquid chromatography (ACQUITY UPLC H-CLASS PLUS/XEVO TQ-XS, Waters). Electron spin resonance (ESR) spectra were accessed by Bruker ESR 5000 technology (Bruker, Germany). Fluorescence images of cells were captured by confocal laser scanning microscope (Nikon C2, Japan). In vivo fluorescence/bioluminescence images were acquired by in vivo imaging systems (IVIS, Perkin Elmer). The analysis of immune cells was performed by flow cytometer (CytoFLEX, Beckman).

**Synthesis of endoplasmic reticulum (ER)-targeted TCBQ/iLD-ER NPs**

The ER-targeting moiety-modified 1,2-distearoyl-sn-glycero-3-phosphoethanolamine-N-[(polyethylene glycol)-5000] (DSPE-PEG-ER) was prepared through the amide reaction between N-tosylethylenediamine and DSPE-PEG-COOH. In a typical procedure, DSPE-PEG-COOH (1 mmol), EDC (2.5 mmol), and NHS (1 mmol) were dissolved in 15 mL of dichloromethane (CH_2_Cl_2_), and then the mixture was reacted at room temperature for 2 h to activate the carboxyl groups. Next, 5 mL of CH_2_Cl_2_ containing ER-targeting N-tosylethylenediamine (1.5 mmol) was added to the above solution. After stirring at room temperature for 12 h, the as-prepared DSPE-PEG-ER was rotary evaporated to remove CH_2_Cl_2_ and re-dispersed in DI water, treated with dialysis in DI water to remove the excess reactants, followed by lyophilization and stored at -20 °C before use.

Subsequently, TCBQ/iLD-ER NPs were fabricated using a nanoprecipitation method. Briefly, 1 mL of dimethyl sulfoxide containing TCBQ (1 mg) and iLD (0.2 mg) was slowly added into 10 mL of aqueous solution containing 20 mg of DSPE-PEG-ER. After stirring at room temperature for 12 h, TCBQ/iLD-ER NPs were collected by ultrafiltration and washed three times with water. The obtained TCBQ/iLD-ER NPs were re-dispersed in DI water and stored at 4 °C before use.

**Generation of hydroxyl radicals (•OH) from H_2_O_2_ by** **TCBQ/iLD-ER NPs**

Aiming to assess the chemodynamic effect of TCBQ/iLD-ER NPs, the generation of reactive oxygen species (ROS) was evaluated by utilizing ABTS as a probe that can be oxidized to generate green oxidized ABTS (ox-ABTS) by ROS. Briefly, ABTS (100 μg/mL), TCBQ/iLD-ER NPs ([TCBQ] = 50 μg/mL), and H_2_O_2_ (100 μM) were sequentially added to buffer solutions with different pH values, and the mixtures were shaken at 37 °C for 5 min. After centrifugation, the absorbance spectra of supernatant were monitored.

To further investigate the species of ROS generated in the TCBQ/iLD-ER NPs + H_2_O_2_ system, ESR analysis was performed by exploiting DMPO as the spin trapping reagent. Specifically, DMPO (10 mg/mL), TCBQ/iLD-ER NPs ([TCBQ] = 50 μg/mL), and H_2_O_2_ (10 mM) were sequentially added to buffer solution with pH 7.4, and ESR spectrum was recorded immediately.

**H_2_O_2_-triggered degradation of TCBQ/iLD-ER NPs**

To verify the H_2_O_2_-triggered degradation of TCBQ/iLD-ER NPs, TCBQ/iLD-ER NPs were incubated with 100 μM H_2_O_2_ in phosphate buffered saline (PBS) solution (pH 7.4) for different periods of time, and then TEM image and dynamic light scattering (DLS) analysis were used to characterize the size change.

The iLD release from TCBQ/iLD-ER NPs was also evaluated. Briefly, TCBQ/iLD-ER NPs were dispersed in 5 mL of buffer solution and transferred to a dialysis bag (MWCO: 3500 Da). Then, the dialysis bag was placed into 45 mL of pH 7.4 buffer solution containing 100 μM H_2_O_2_. At appropriate time points, 1 mL of buffer solution was taken out and replaced with the same volume of buffer solution. The cumulative iLD release was quantified by ultra performance liquid chromatography (UPLC).

**ER-targeting ability of TCBQ/iLD-ER NPs**

4T1 cells were seeded in confocal dishes and cultured for 24 h. After incubation with Cy5-labeled TCBQ/iLD-ER NPs for 4 h, 4T1 cells were stained with ER-tracker green (1 μM) and Hoechst 33342 (5 μg/mL) for another 20 min, and then analyzed by the CLSM.

**In vitro LDs analysis**

4T1 cells were seeded in confocal dishes and incubated overnight. After 12 h of incubation with iLD, TCBQ, TCBQ/iLD NPs, or TCBQ/iLD-ER NPs ([iLD] = 4 μg/mL, [TCBQ] = 20 μg/mL), the cells were stained with Nile red (a fluorescent dye for LDs, 1 μM) and Hoechst 33342 (5 μg/mL) for 20 min prior to visualization by the CLSM.

**In vitro ROS generation**

4T1 cells were seeded in confocal dishes and incubated with iLD, TCBQ, TCBQ/iLD NPs, or TCBQ/iLD-ER NPs ([iLD] = 4 μg/mL, [TCBQ] = 20 μg/mL) for 4 h. Subsequently, the cells were stained with DCFH-DA (10 μM) and Hoechst 33342 (5 μg/mL) for 20 min before being observed with the CLSM.

**LPO induced by TCBQ/iLD-ER NPs**

4T1 cells were cultured with iLD, TCBQ, TCBQ/iLD NPs, or TCBQ/iLD-ER NPs ([iLD] = 4 μg/mL, [TCBQ] = 20 μg/mL) for 6 h. Next, the cells were stained with C11-BODIPY^581/591^ (10 μM) and Hoechst 33342 (5 μg/mL) for another 20 min, and the variation of fluorescence from red to green was monitored.

The production of LPO toxic byproducts 4‑HNE or MDA was evaluated with 4-HNE ELISA kit or MDA assay kit, respectively. 4T1 cells were seeded in 6-well plates and incubated for 12 h. Then, the cells were treated with iLD, TCBQ, TCBQ/iLD NPs, or TCBQ/iLD-ER NPs ([iLD] = 4 μg/mL, [TCBQ] = 20 μg/mL) for 24 h. Ultimately, the 4T1 cells and cell culture mediums were collected, and the production of 4-HNE or MDA was determined by corresponding kits according to the manufacturer’s protocols.

**Evaluate ER stress by** **Western blot**

4T1 cells were seeded in 6-well plates and treated with iLD, TCBQ, TCBQ/iLD NPs, or TCBQ/iLD-ER NPs ([iLD] = 4 μg/mL, [TCBQ] = 20 μg/mL) for 24 h. Then, total cellular proteins were extracted from treated cells and subjected to western blotting analysis. Following electrophoretic separation and membrane transfer, the immobilized proteins were probed with anti-GRP78 antibody or anti-CHOP antibody at 4 °C overnight, subsequently incubated with horseradish peroxidase-conjugated secondary antibody at room temperature for 1 h, and ultimately visualized through chemiluminescent detection.

**Chemodynamic cytotoxicity of TCBQ/iLD-ER NPs**

CCK-8 assay was applied to quantitatively assess the chemodynamic efficacy of TCBQ/iLD-ER NPs. 4T1 cells were seeded in 96-well plates and incubated with different concentrations of iLD, TCBQ, TCBQ/iLD NPs, or TCBQ/iLD-ER NPs for 24 h. Afterward, the medium was replaced with serum-free medium containing 10 vol% of CCK-8, and incubated for another 1 h at 37 °C. Then, absorbance at 450 nm was monitored by a microplate reader to compare the cell viabilities and assess the chemodynamic cytotoxicity of TCBQ/iLD-ER NPs against cancer cells.

Calcein-AM/PI double staining assay was used to intuitively estimate the cancer cell killing effect of TCBQ/iLD-ER NPs. 4T1 cells were seeded in confocal dishes and incubated overnight. Next, after exposure to iLD, TCBQ, TCBQ/iLD NPs, or TCBQ/iLD-ER NPs ([iLD] = 4 μg/mL, [TCBQ] = 20 μg/mL) for 24 h, the cells were stained with calcein-AM (1 μM) and PI (2 μM) for 20 min. Subsequently, the CLSM images were recorded.

Annexin V-FITC/PI apoptosis detection assay to examine the TCBQ/iLD-ER NPs-induced cell apoptosis via FCM. 4T1 cells were seeded in 6-well dishes and treated with iLD, TCBQ, TCBQ/iLD NPs, or TCBQ/iLD-ER NPs ([iLD] = 4 μg/mL, [TCBQ] = 20 μg/mL) for 24 h. Subsequently, the cells were stained with an annexin V-FITC/PI kit according to the manufacturer’s protocol, and the quantitative analysis of apoptotic cells was evaluated by FCM.

**Detection of ATP or HMGB1 release induced by TCBQ/iLD-ER NPs**

4T1 cells were seeded in 6-well plates and treated with iLD, TCBQ, TCBQ/iLD NPs, or TCBQ/iLD-ER NPs ([iLD] = 4 μg/mL, [TCBQ] = 20 μg/mL) for 24 h. Ultimately, the cell culture mediums were collected, and the released ATP or HMGB1 was detected by ATP assay kit or HMGB1 ELISA kit according to the manufacturer’s protocols.

**Immunofluorescence staining of CRT or HMGB1**

4T1 cells were seeded in confocal dishes and incubated with iLD, TCBQ, TCBQ/iLD NPs, or TCBQ/iLD-ER NPs ([iLD] = 4 μg/mL, [TCBQ] = 20 μg/mL) for 24 h. Next, the cells were fixed with 4% paraformaldehyde (PFA) for 10 min and permeabilized with 0.1% Triton X-100 for 10 min at room temperature. After being washed three times with PBS, the cells were blocked with 10% goat serum for 30 min at room temperature. Following that, the cells were incubated with anti-CRT or anti-HMGB1 rabbit monoclonal antibody overnight at 4 ℃, and then washed with PBS three times. Subsequently, the cells were stained with goat anti-rabbit IgG H&L (Alexa Fluor^®^ 568, 1:1000 dilution) and Hoechst 33342 (5 μg/mL) for another 1 h at 37 ℃, and the CLSM images were recorded.

**Co-localization analysis of LDs and CRT**

4T1 cells were seeded in confocal dishes and treated with iLD, TCBQ, TCBQ/iLD NPs, or TCBQ/iLD-ER NPs ([iLD] = 4 μg/mL, [TCBQ] = 20 μg/mL) for 24 h. Next, the cells were incubated with the anti-CRT rabbit monoclonal antibody overnight at 4 ℃ and followed by Alexa Fluor 488-conjugated secondary antibody for another 1 h at 37 ℃. After staining with Nile red (1 μM) and Hoechst 33342 (5 μg/mL) for another 20 min, the co-localization analysis was measured by CLSM.

**In vitro dendritic cells (DCs) maturation**

Bone-marrow-derived DCs were collected from bone marrow of 6-8 weeks BALB/c mice according to previous established approach.^[1, 2]^ Concretely, bone marrow was acquired by flushing the femur and tibia with PBS. After the lysis of red blood cells, the remaining cells were inoculated into a cell culture dish with 10 mL RPMI 1640 medium (10% fetal bovine serum, 1% penicillin-streptomycin, 20 ng/mL of GM-CSF, and 20 ng/mL of IL-4). On the 7th day, the non-adherent and loosely adherent immature DCs were collected for further use. Residues of 4T1 cells after treatment with iLD, TCBQ, TCBQ/iLD NPs, or TCBQ/iLD-ER NPs ([iLD] = 4 μg/mL, [TCBQ] = 20 μg/mL) were co-cultured with immature DCs in the transwell system for 24 h. Subsequently, DCs were collected and stained with anti-CD11c-FITC, anti-CD80-PE, and anti-CD86-APC monoclonal antibodies for 20 min prior to analysis by FCM. Also, the culture supernatant of DCs was collected to measure the secretion of immunostimulatory cytokines, including TNF-α, IL-6, IL-12p70, and IL-10, by corresponding ELISA kits according to the manufacturer’s protocols.

**In vitro T cells activation**

CD3^+^ T lymphocytes were isolated from the spleen of 6-8 weeks BALB/c mice and purified using the MagniSor mouse CD3 positive selection kit. Purified T cells were co-cultured with pre-educated CD11c^+^ DCs at a ratio of 5:1 for 48 h. For the assessment of T cell activation, cell surface stained with anti-CD3ε-FITC, anti-CD8a-PE, and anti-CD4-APC monoclonal antibodies for FCM analysis.

**In vitro T-cell killing assay**

Pre-activated T cells were co-cultured with 4T1 cells at a ratio of 10:1 for 48 h. Then, the 4T1 cells were collected and stained with an annexin V-FITC/PI kit according to the manufacturer’s protocol, and the quantitative analysis of apoptotic cells was evaluated by FCM.

**Animal tumor models**

For 4T1 tumor-bearing mice models, female BALB/c mice (6-8 weeks) were subcutaneously injected with 1×10^6^ 4T1 cells into the right flank. 7 days later, the tumor volumes reached approximately 80 mm^3^, and the mice were randomly allocated into five groups (5 mice per group) and then intravenously injected with PBS, iLD, TCBQ, TCBQ/iLD NPs, or TCBQ/iLD-ER NPs ([iLD] = 1 mg/kg, [TCBQ] = 5 mg/kg) at days 0, 2, 4, and 6. The tumor volumes and body weights were recorded every other day. Tumor volume = (tumor length) × (tumor width)^2^/2. The mice were euthanized for histological examination after 14 days of treatment.

For 4T1 bilateral tumor-bearing mice models, female BALB/c mice (6-8 weeks) were subcutaneously injected with 1×10^6^ 4T1 cells into the right flank (primary tumor), followed by the subcutaneous injection of 1×10^6^ 4T1 cells into the left flank (distant tumor) 4 days later. Another 5 days later, the primary and distant tumor volumes reached approximately 100 mm^3^ and 50 mm^3^, respectively, and the mice were randomly allocated into five groups (8 mice per group) and then intratumorally injected with PBS, iLD, TCBQ, TCBQ/iLD NPs, or TCBQ/iLD-ER NPs ([iLD] = 0.2 mg/kg, [TCBQ] = 1 mg/kg) into primary tumor sites at days 0, 2, 4, and 6. The tumor volumes and body weights were recorded every other day.

For lung metastasis models, female BALB/c mice (6-8 weeks) were subcutaneously injected with 1×10^6^ 4T1 cells into the right flank. 5 days later, the tumor volumes reached approximately 50 mm^3^, and the mice were randomly allocated into five groups (3 mice per group) and then intratumorally injected with PBS, iLD, TCBQ, TCBQ/iLD NPs, or TCBQ/iLD-ER NPs ([iLD] = 0.2 mg/kg, [TCBQ] = 1 mg/kg) into tumor sites at days 0, 2, 4, and 6. On the 7th day, the treated 4T1 tumor-bearing mice were intravenously injected with 1×10^5^ 4T1-Luc cells. The metastasis of 4T1-Luc cells in mice was monitored by IVIS, and body weights were recorded every other day. Another 14 days later, the lungs were harvested from each group and fixed with 4% PFA for metastatic nodule counting and further studied by hematoxylin and eosin (H&E) staining assay.

**In vivo immunity activation**

To systematically investigate the antitumor immune responses in vivo, tumors, tumor-draining lymph nodes (TDLNs), spleens, and serum of post-treatment mice were harvested. The tumors, TDLNs, and spleens were made into a single-cell suspension according to the specified procedure.

To assess the maturation status of DCs in TDLNs, the single-cell suspensions of distant TDLNs were stained with anti-CD11c-FITC, anti-CD80-PE, and anti-CD86-APC monoclonal antibodies for FCM analysis.

To analyze CD8^+^ and CD4^+^ T cells in tumor tissues, the single-cell suspensions of distant tumors were stained with anti-CD3ε-FITC, anti-CD8a-PE, and anti-CD4-APC monoclonal antibodies for FCM analysis.

To analyze exhausted T cells in tumor tissues, the single-cell suspensions of distant tumors were stained with anti-CD3ε-FITC, anti-CD8a-PE, and anti-TIM-3-APC monoclonal antibodies for FCM analysis.

To evaluate splenic activated CD8^+^ and CD4^+^ T cells, the single-cell suspensions of spleens were stained with anti-CD3ε-FITC, anti-CD8a-PE, and anti-CD4-APC monoclonal antibodies for FCM analysis.

To analyze memory T cells, the single-cell suspensions of spleens were stained with anti-CD3ε-FITC, anti-CD8a-PE, anti-CD44-APC, and anti-CD62L-APC/Cyanine7 monoclonal antibodies for FCM analysis.

To detect the secretion of immunostimulatory cytokines in serum, the levels of TNF-α, IL-6, IL-12p70, and IL-10 were determined by corresponding ELISA kits according to the manufacturer’s protocols.

**Figure S1.** Synthesis route of DSPE-PEG-ER.

**Figure S2.** ^1^H NMR spectra of DSPE-PEG-ER, DSPE-PEG-COOH, and N-tosylethylenediamine in chloroform-d (CDCl_3_).

**Figure S3.** ^13^C NMR spectra of DSPE-PEG-ER, DSPE-PEG-COOH, and N-tosylethylenediamine in CDCl_3_.

**Figure S4.** Zeta potential analysis of DSPE-PEG-COOH and DSPE-PEG-ER. n = 3. Data are presented as mean ± SD.

**Figure S5.** UV-Vis absorption spectra of iLD, iLD NPs, TCBQ, TCBQ NPs, TCBQ/iLD NPs, or TCBQ/iLD-ER NPs (iLD NPs and TCBQ NPs were synthesized using DSPE-PEG-COOH as encapsulation matrix).

**Figure S6.** DLS size distribution of a) TCBQ/iLD NPs and b) TCBQ/iLD-ER NPs.

**Figure S7.** Zeta potential analysis of TCBQ/iLD NPs and TCBQ/iLD-ER NPs. n = 3. Data are presented as mean ± SD.

**Figure S8.** Mechanism diagram of the reaction between TCBQ and H_2_O_2_.

**Figure S9.** DLS size distribution of TCBQ/iLD-ER NPs after incubation with H_2_O_2_ at pH 7.4 for different periods of time.

**Figure S10.** CLSM images of 4T1 cells after incubation with Cy5-labeled TCBQ/iLD-ER NPs for different periods of time.

**Figure S11.** CLSM images of 4T1 cells incubated with Cy5-labeled TCBQ/iLD NPs or TCBQ/iLD-ER NPs (red channel) and Mito-tracker (green channel). The corresponding Pearson’s correlation coefficients (R) were calculated. Fluorescence intensity profiles were along the corresponding white dashed arrows (25 μm).

**Figure S12.** FCM histogram of LDs levels in 4T1 cells by Nile red staining after incubation with different formulations for 12 h.

**Figure S13.** •OH-specific O27 probe staining of 4T1 cells after incubation with different formulations for 4 h.

**Figure S14.** C11-BODIPY^581/591^ staining of 4T1 cells after incubation with different formulations for 6 h.

**Figure S15.** 4-HNE and MDA levels in the supernatant of 4T1 cells after 24 h of incubation with different formulations. n = 3. Data are presented as mean ± SD. **p < 0.01, ***p < 0.001.

**Figure S16.** Uncropped Western blot images of Figure 2h.

**Figure S17.** Lactate dehydrogenase (LDH) release from 4T1 cells after treatment with different formulations for 24 h (compared with cells exposed to the LDH release reagent). n = 3. Data are presented as mean ± SD. **p < 0.01, ***p < 0.001.

**Figure S18.** Calcein-AM/PI co-staining of 4T1 cells after treatment with different formulations for 24 h.

**Figure S19.** FCM analysis of 4T1 cells apoptosis after exposure to different formulations for 24 h.

**Figure S20.** CLSM images displaying CRT exposure on the surface of 4T1 cells after incubation with different formulations for 24 h.

**Figure S21.** Co-localization analysis of LDs (red channel) and CRT (green channel) in 4T1 cells after incubation with different formulations for 24 h.

**Figure S22.** CLSM images displaying intracellular HMGB1 of 4T1 cells after incubation with different formulations for 24 h.

**Figure S23.** a) UV-vis spectra of erythrocytes exposed to DI water or different concentrations of TCBQ/iLD-ER NPs in PBS. b) The percentages of hemolytic reaction in response to different concentrations of TCBQ/iLD-ER NPs (Inset: photograph of centrifuge tubes containing the supernatant from erythrocytes exposed to DI water or different concentrations of TCBQ/iLD-ER NPs in PBS). n = 3. Data are presented as mean ± SD.

**Figure S24.** Ex vivo fluorescence image of major organs and tumor harvested from 4T1 tumor-bearing mice at 24 h post-injection of Cy5-labeled TCBQ/iLD-ER NPs.

**Figure S25.** Individual tumor growth curves of 4T1 tumor-bearing mice post-injection with PBS, iLD, TCBQ, TCBQ/iLD NPs, or TCBQ/iLD-ER NPs.

**Figure S26.** Representative CD8 and CD4 immunofluorescence staining of tumor slices harvested from 4T1 tumor-bearing mice in various groups after 14 days of treatment.

**Figure S27.** Body weight curves of 4T1 tumor-bearing mice post-injection with different formulations. n = 5. Data are presented as mean ± SD.

**Figure S28.** Blood biochemistry analysis in mice intravenously injected with PBS, iLD, TCBQ, TCBQ/iLD NPs, or TCBQ/iLD-ER NPs (day 14). ALT, alanine transferase; AST, aspartate transferase; ALP, alkaline phosphatase; BUN, blood urea nitrogen; CRE, creatinine. n = 3. Data are presented as mean ± SD.

**Figure S29.** Representative H&E staining of major organs slices harvested from 4T1 tumor-bearing mice in various groups after 14 days of treatment.

**Figure S30.** Individual tumor growth curves of 4T1 bilateral tumor-bearing mice post-injection with PBS, iLD, TCBQ, TCBQ/iLD NPs, or TCBQ/iLD-ER NPs.

**Figure S31.** Representative CD8 and CD4 immunofluorescence staining of tumor slices harvested from 4T1 bilateral tumor-bearing mice in various groups after 14 days of treatment.

**Figure S32.** Body weight curves of 4T1 bilateral tumor-bearing mice post-injection with different formulations. n = 8. Data are presented as mean ± SD.

**Figure S33.** Representative H&E staining of major organs slices harvested from 4T1 bilateral tumor-bearing mice in various groups after 14 days of treatment.

**Figure S34.** FCM histogram of exhausted T cells (TIM-3^+^) within distant tumors from mice after treatment with different formulations (gating on CD3^+^CD8^+^).

**Figure S35.** a) Surface expression of CD8 and CD4 on T cells within spleens harvested from 4T1 bilateral tumor-bearing mice after treatment with different formulations quantitatively analyzed by FCM (gating on CD3^+^) and b,c) corresponding quantification histogram. n = 3. Data are presented as mean ± SD. **p < 0.01, ***p < 0.001.

**Figure S36.** FCM analysis of effector memory T cells (CD44^+^CD62L^−^) and central memory T cells (CD44^+^CD62L^+^) in spleens harvested from 4T1 bilateral tumor-bearing mice after treatment with different formulations (gating on CD3^+^CD8^+^).

**References**

1. Y. Liu, W. Zhen, Y. Wang, S. Song, and H. Zhang, “Na_2_S_2_O_8_ Nanoparticles Trigger Antitumor Immunotherapy through Reactive Oxygen Species Storm and Surge of Tumor Osmolarity,” *Journal of the American Chemical Society* 142, no. 52 (2020): 21751-21757. <https://doi.org/10.1021/jacs.0c09482>.

2. S. Singh, A. Tehseen, S. Dahiya, Y. J. Singh, R. Sarkar, and S. Sehrawat, “Rab8a Restores Diverse Innate Functions in CD11c^+^CD11b^+^ Dendritic Cells from Aged Mice,” *Nature Communications* 15, no. 1 (2024): 10300. <https://doi.org/10.1038/s41467-024-54757-2>.
